# Treatment of pediatric supracondylar humerus fractures accompanied with pink pulseless hands

**DOI:** 10.1186/s12891-020-03877-z

**Published:** 2021-01-06

**Authors:** Li-wei Xie, Juan Wang, Zhi-qiang Deng

**Affiliations:** 1 Department of Pediatric Orthopedics, Sichuan Provincial Orthopedics Hospital, Chengdu, Sichuan China; 2 Department of Geriatrics, Chengdu Shuang-nan Hospital, Chengdu, Sichuan China

**Keywords:** Supracondylar humeral fracture, Vascular compromise, Pink pulseless hand, Children

## Abstract

**Background:**

The optimal treatment for pediatric supracondylar humeral fractures accompanied with a pink pulseless hand is controversial. Some clinicians recommend close observation after closed reduction and percutaneous pinning of the fractures, while some recommend surgical exploration if the radial pulse is unpalpable. The present study aimed to analyze the benefits and outcomes of close observation for treating pediatric supracondylar humeral fractures with a pink pulseless hand.

**Methods:**

Thirteen consecutive children presenting with a pink pulseless hand following supracondylar humeral fracture were enrolled in this study. Preoperative and postoperative color-flow Duplex ultrasound detection was used to assess brachial artery compromise in most cases. Urgent closed reduction and percutaneous pinning of the fractures were attempted first. Close observation was carried out when the hand was pink and pulseless with an absent radial pulse.

**Results:**

Preoperative color-flow Duplex ultrasound showed no disruption of the brachial artery in cases detected. Compression of the artery by the proximal fragment was observed in most cases, with one case of entrapment of the artery between fragments, and thrombus considered in two cases. All cases underwent urgent surgery, after which nine experienced immediate return of the radial pulse. The remaining four without a palpable pulse were managed with close observation and no deterioration of the vascular status was observed; therefore, no surgical exploration was performed. Postoperative color-flow Duplex ultrasound revealed continuity of the artery and rich collateral circulation. Patients completed an average of 4.5 years of follow-up, during which no major complications occurred. All patients achieved excellent limb function.

**Conclusions:**

Our study demonstrates that close observation after urgent closed reduction and percutaneous pinning is a sufficient approach for the treatment of pediatric supracondylar humeral fractures accompanied with a pink pulseless hand. Surgical exploration is not necessary as long as the hand is warm and well perfused. Color-flow Duplex ultrasound is beneficial for assessing vascular compromise and determining treatment strategies.

## Background

Supracondylar humerus fractures (SHFs) are the most common fractures in children and account for approximately 50–70% of pediatric elbow fractures [1, 2]. Compromised vasculature occurs in 2.6–20% of cases of displaced SHFs in children [3–6], with two kinds of brachial artery injuries reported to be associated with SHFs: those presenting with a pale pulseless hand and those with a pink pulseless hand (PPH) which is well perfused without a palpable radial pulse [6–8]. While vascular compromise in elderly patients with SHFs is rare due to osteoporosis and low-energy trauma compared with pediatric patients, open reduction and plate fixation was recommended for satisfactory outcomes [9]. In pediatric patients, urgent closed reduction and percutaneous pinning (CRPP) is the primary treatment in both situation, and vascular exploration is often required in the case of a pale pulseless hand [6–8, 10, 11]. However, the optimal treatment for a PPH in terms of whether to perform immediate vascular exploration or manage with close observation is controversial in cases where the radial pulse is still not palpable after CRPP [10, 11].

Some clinicians recommend aggressive surgical exploration and vascular reconstruction due to the concern that delaying reconstruction of the artery will lead to compartment syndrome, ischemic contracture, retarded development of the limb, cold intolerance, and so on [3, 8, 11–15]. Blakey et al. reported a high incidence of compartment syndrome associated with a PPH following SHFs [16], and the incidence has been reported to increase from 0.2–4.5% in cases with co-existing neurovascular compromise [17].

However, conservative management (close observation) is recommended as long as the hand is warm and well-perfused, with the reasoning that collateral circulation of the elbow joint will provide sufficient blood supply for the limb, and vascular exploration such only be carried out when deterioration of the circular status of the limb occurs [4–7, 12, 18–23]. Choi et al. reported that, among patients treatment for absent distal pulse following displaced SHFs, CRPP resulted in a good rate of return of palpable pulse after surgery or achievement of a pulseless but well-perfused status [5]. Good outcomes were achieved without surgical exploration and no patients developed compartment syndrome. Scannell et al. described that CRPP for the treatment of perfused pulseless hand following displaced SHFs resulted in an immediate return of palpable pulse in 20% of patients, with normal radial pulse returning in all patients eventually [18]. However, patent brachial arteries occurred in almost 75% of patients and brachial artery occlusion in 20%; collateral vessels were also found, severe arterial stenosis occurred in one patient, and cold intolerance during participation in ice hockey was reported in one patient. At an average of 20 months follow-up, there were no differences in arm circumference or length, elbow motion, muscle endurance, or grip strength between the injured and uninjured sides. The traditional conservative strategy of close observation has been used for SHFs since 1950s and relies on collateral circulation of the elbow joint [11, 20, 24–26]. Studies have demonstrated that the limb can kept alive with ligation of the injured brachial artery [27–29]. Strong collateral circulation has been identified using arteriography or ultrasound around the elbow after SHFs [20].

However, there have been few reports of the use color-flow Duplex ultrasound (CFDU) for the assessment of brachial artery compromise and the status of collateral circulation before and after surgical treatment of SHFs [11, 18, 30, 31]. The present study aimed to address this gap in the knowledge by analyzing the outcomes of conservative management (close observation) for pediatric SHFs with a PPH, and to evaluate the utility of CFDU for assessing lesions of the brachial artery.

## Methods

### Patients

We retrospectively reviewed all patients aged 14 years or below who were admitted for displaced SHFs from May 2014 to November 2018 at our pediatric orthopedic center. All patients underwent a thorough physical examination upon presentation at the emergency room to identify potential neurovascular compromise. Those with a pink pulseless hand which means brachial artery compromise would be enrolled in the study. Those without vascular compromise were excluded. This study and all its protocols were approved by the institutional review board.

### Treatment strategy

Once vascular compromise was confirmed, the arm was immobilized with a temporary posterior cast in a position of 30°–40° of elbow flexion and CFDU for vascular detection ordered with an urgent CRPP arranged at the same time. But the CFDU detection failed in three cases because of noncooperation of the patients. All patients underwent CRPP under general anesthesia guided by fluoroscopy after which the arm was immobilized in 70° of elbow flexion with a posterior long-arm cast. Relaxant was administered to guarantee sufficient muscle relaxation and traction to ensure that separation of fragments was achieved, confirmed by anterior posterior fluoroscopy, to avoid aggravating the injured artery. Patients were closely observed for vascular status including monitoring of temperature, color, capillary refill, pain, palsy, and return of distal radial pulse using a portable ultrasound device on the pediatric ward. Further CFDU was carried out 1–2 days postoperatively to reassess the brachial artery and collateral circulation in most cases. The cast was removed 4 weeks postoperatively and functional exercises implemented. Pins were removed 1 week later. All patients were followed up and complications were recorded.

## Results

In total, 1267 patients underwent CRPP for SHFs at our center during the study period. Of these, 1079 were classified as Gartland Type III fractures, 13 of which (eight boys and five girls) presented with a PPH and were finally enrolled in the study. Open fractures were excluded. The average age of participants was 6 years (range: 2–11 years). All 13 cases were severe closed fractures with distal fragment posterolateral and posteromedial displacement in nine and four cases respectively. Physical examination revealed eight fractures to be accompanied with neurologic deficits: seven had median-nerve palsy (four had isolated anterior-interosseous-nerve palsy, three had only median-sensory-nerve palsy), one had radial-sensory-nerve palsy, and one had ulnar-motor-nerve palsy (Table 1). The average time from injury to operation was 12 hours (range: 1.5–48 hours), the average time of operation was 43 minutes (range: 25–65 minutes), and the average length of hospital stay was 5.4 days (range: 2–10 days).

**Table 1:** Patient Demographics, Injury Characteristics, Treatment, and Outcome

Case	Age (y)	Sex	Type of Fracture By Gartland Classification	Direction of Distal Fragment	Nerve Injury	Time From Injury to Procedure(h)	Procedure	Postoperative Vascular Status	Recovery of Radial Pulse	Final Follow-up
1	6	M	III(closed)	Posteromedial	None	1.5	CRPP	Well-perfused No palpable pulse	54hrs Post-op	Normal
2	4.3	M	III(closed)	Posterolateral	None	10.5	CRPP	Well-perfused Palpable pulse	Immediately post-op	Normal
3	6.7	F	III(closed)	Posteromedial	M(s)	6	CRPP	Well-perfused Palpable pulse	Immediately post-op	Normal
4	11.4	M	III(closed)	Posterolateral	M(s)	8	CRPP	Well-perfused Palpable pulse	Immediately post-op	Normal
5	9.6	M	III(closed)	Posterolateral	M&R(s)	6	CRPP	Well-perfused No palpable pulse	16hrs Post-op	Normal
6	4.3	M	III(closed)	Posterolateral	M(m)	8	CRPP	Well-perfused No palpable pulse	1 m Post-op	Normal
7	6.3	F	III(closed)	Posterolateral	U(m)	48	CRPP	Well-perfused Palpable pulse	Immediately post-op	Normal
8	4.4	M	III(closed)	Posterolateral	M(s)	45	CRPP	Well-perfused Palpable pulse	Immediately post-op	Normal
9	2.5	F	III(closed)	Posteromedial	None	5.5	CRPP	Well-perfused Palpable pulse	Immediately post-op	Normal
10	5.2	F	III(closed)	Posteromedial	M(m)	4	CRPP	Well-perfused Palpable pulse	Immediately post-op	Normal
11	6	F	III(closed)	Posterolateral	M(m)	3	CRPP	Well-perfused Palpable pulse	Immediately post-op	Normal
12	4.5	M	III(closed)	Posterolateral	None	6	CRPP	Well-perfused Palpable pulse	Immediately post-op	Normal
13	8.3	M	III(closed)	Posterolateral	None	5	CRPP	Well-perfused No palpable pulse	6ws Post-op	Normal

*M* Median nerve, *R* Radial nerve, *U* Ulnar nerve, *m* Motor nerve, *s* Sensory nerve. *CRPP* Closed reduction and percutaneous pinning

Preoperative CFDU, carried out in ten of the thirteen patients, revealed compression of the brachial arteries by the tip of the proximal fragments in most cases, resulting in narrowing of the inner diameter of the vessel and decreased blood stream velocity. One case exhibited entrapment of the brachial artery between fragments. Thrombus formation was considered at the fracture site in two cases and collateral circulation was detected around the elbow joint in three. No disruption or active bleeding of the brachial artery was found in cases underwent detection. The blood flow velocities of the radial and ulnar arteries were decreased, demonstrating a venous spectrum (Table 2).

**Table 2:** Type of Injury of the Brachial Artery, Characteristics of Pre- and Postoperation CFDU

Case	Type of Brachial Artery Lesion by Pre-op CFDU Detection	Preoperation CFDU	Postoperation CFDU	Collateral Circulation
1	Unclear	No	No	Unclear
2	Compressed	Narrowed lumen of distal BA Slowed blood velocity of BA, RA, UA VB = 58.4 cm/s; VR = 20.2 cm/s; VU = 15.9 cm/s	Narrowed lumen Slowed blood velocity of BA, RA, UA VB = 156 cm/s; VR = 84.1 cm/s; VU = 34.3 cm/s	Undescribed
3	Compressed	Narrowed lumen of distal BA Slowed blood velocity of B, R, U artery	Narrowed lumen Slowed blood velocity of BA, RA, UA VB = 17.4 cm/s; VR = 19.3 cm/s; VU = 25.5 cm/s	Detected
4	Compressed	Narrowed lumen of distal BA Slowed blood velocity of BA, RA, UA VB = 20.8 cm/s; VR = 38.5 cm/s; VU = 27.6 cm/s	No	Undescribed
5	Unclear	No	Hypoecho and no detected blood flow signal of distal BA and proximal RA thrombus is considered	Undescribed
6	Entrapment	Entrapment of BA between fragments Star or dot in shape signal of distal BA Slowed blood velocity of RA, UA VR = 10 cm/s; VB = 10 cm/s thrombus is considered	First time postoperation: VU star or dot in shape signal of distal BA VR = 19.7 cm/s; VB = 20.6 cm/s; collateral circulation is plentiful	Detected
			Second time postoperation: VU star or dot in shape signal of distal BA Slowed blood velocity of RA, UA VR = 38 cm/s; VB = 42 cm/s	
			Third time postoperation: Narrowed lumen of distal BA Slowed blood velocity of BA, RA, UA VB = 36.0 cm/s; VR = 13.4 cm/s; VU = 19.7 cm/s Collateral circulation is less than previous A change after thrombus is considered	
7	Compressed	Narrowed lumen of distal BA Slowed blood velocity of BA	No	Undescribed
8	Unclear	No	No	Unclear
9	Compressed	Narrowed lumen of distal BA undetected signal of distal BA Slowed blood velocity of RA, UA	No	Undescribed
10	Compressed	Narrowed lumen of distal BA Slowed blood velocity of BA, RA, UA	Narrowed lumen of distal BA Slowed blood velocity of BA, RA, UA VB = 6.9 cm/s; VR = 24.4 cm/s; VU = 17.8 cm/s	Undescribed
11	Compressed	Narrowed lumen of distal BA Slowed blood velocity of BA, RA, UA	No	Undescribed
12	Compressed	Narrowed lumen of distal BA undetected signal of distal BA Slowed blood velocity of RA, UA VR = 11.8 cm/s; VU = 10.6 cm/s A branch of collateral circulation extend to UA	Artery of upper limb is normal VB = 109 cm/s; VR = 55.4 cm/s; VU = 66.7 cm/s	Detected
13	Compressed	Narrowed lumen of distal BA Slowed blood velocity of BA, RA, UA	Narrowed lumen of distal BA Slowed blood velocity of BA, RA, UA	Undescribed

*CFDU* Color-flow Duplex Ultrasound, *BA* Brachial artery, *RA* Radial artery, *UA* Ulnar artery, *VB* Velocity of distal brachial artery, *VR* Velocity of distal radial artery, *VU* Velocity of distal ulnar artery

**Fig. 1:**
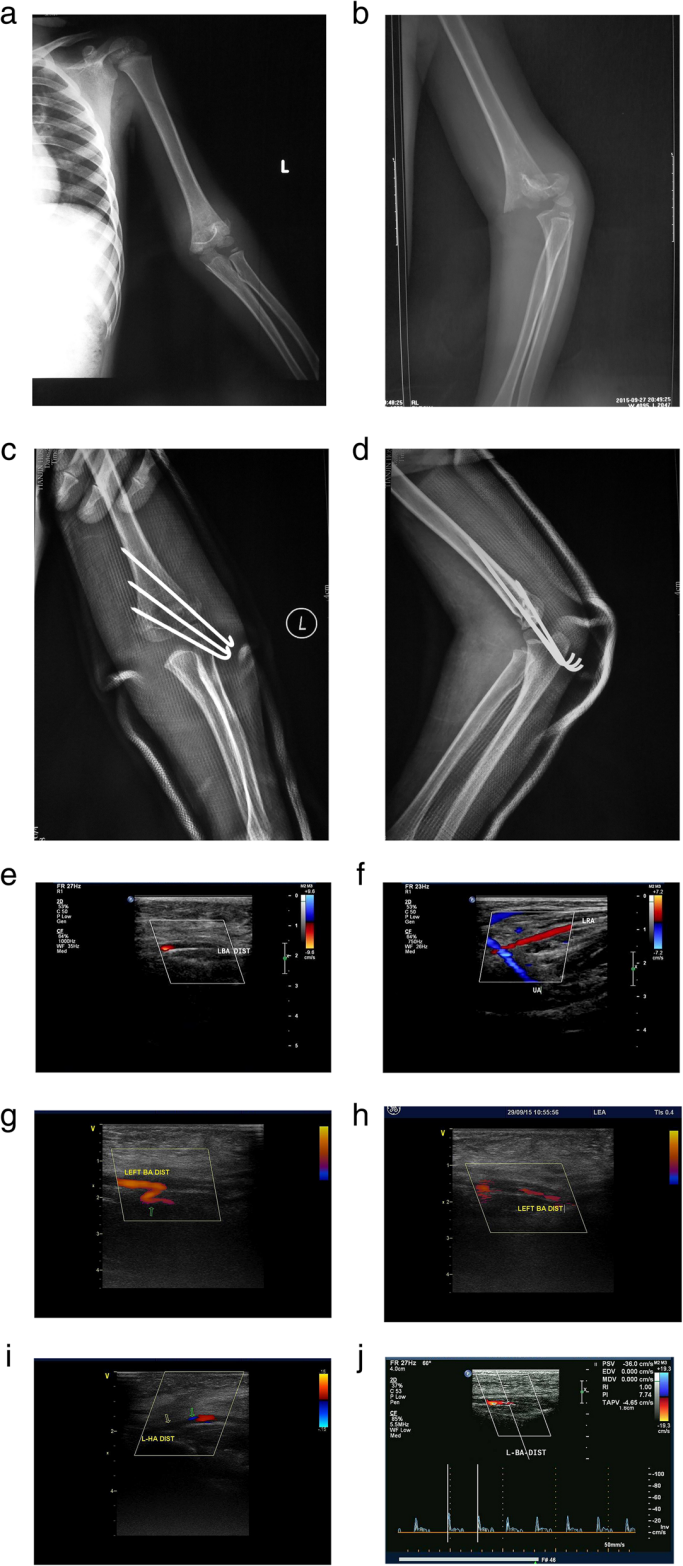
**a** Gartland type III fracture treated with CRPP, following by a pink pulseless hand treated with close observation. **a-d** Pre- and postoperative films of the fracture. **e-f** Preoperative CFDU showed little blood stream through the distal part of brachial artery but there was blood flow in the radial and ulnar artery. **g-h** The first time postoperative CFDU showed that collateral circulation (green arrow) showed up and blood flow can be detected in the distal part of brachial artery, and thrombus formation is considered. **i-j** The second and third time postoperative CFDU showed the position of thrombus(yellow arrow) and a gradual recovery of the blood flow in the distal part of brachial artery(green arrow)

After CRPP, nine patients experienced immediate return of a palpable radial pulse although this was weaker than that of the contralateral side. This became normal in 2–9 hours postoperatively. The remaining four patients did not have a palpable radial pulse but a PPH was observed closely. Of these, palpable radial pulse returned in two within 16 and 54 hours. Radial pulse did not return before discharge for two patients; however, hands remained warm and well perfused and a radial pulse returned in 6 weeks in one patient and 1 month in the other. Seven patients underwent postoperative CFDU detection to reassess vascular status which revealed continuity of the arteries with no rupture or transection detected. One patient underwent CFDU four times which revealed a gradual increase of the blood flow velocity of the brachial, radial, and ulnar arteries and a gradual decease of collateral circulation (Fig. 1). No patient developed compartment syndrome or required surgical exploration.

The average follow-up period was 4.5 years. All thirteen patients experienced full recovery of the nerve injury with a palpable distal radial pulse as strong as the contralateral side. All patients had normal function of the elbow joints and no obvious scars, cosmetic deformities, ischemic contractures, disturbance of growth, exercise-induced ischemia, cold intolerance, or other abnormalities was observed.

## Discussion

The decision of whether to undertake surgical exploration for SHFs is a subject of some debate presenting with a PPH [11, 12]. Some clinicians recommend aggressive exploration and vascular reconstruction [3, 8, 11, 13–15] while others recommend close observation after urgent CRPP [4–7, 12, 18–23]. The present study demonstrates the benefits of a strategy of close observation following CRPP for the treatment of SHFs presenting with a PPH.

Many studies have described good results of this strategy and few complications [5, 6, 18, 22, 32]. Louahem et al. treated 68 patients with vascular compromise (63 of whom with pulseless perfused hand) with CRPP followed by close observation. Forty-two patients had a palpable radial pulse immediately after CRPP, eighteen experienced return of radial pulse from a few hours to 11 days postoperatively. Three patients required immediate surgical exploration for ischemic signs after unsuccessful reduction, which revealed incarceration of the brachial artery between fragments. With an average of 8.4 years of follow-up, all patients achieved normal circulatory status including a palpable radial pulse. No complications such as limb length discrepancy, cold intolerance, or claudication were observed [6].

Our results agree well with those of the above described studies; all 13 patients who presented with a PPH underwent urgent CRPP. Immediate return of radial pulse was observed in most cases, good capillary refill was detected in those where radial pulse was not palpable, and all patients had a palpable radial pulse at the final follow-up with no complications observed.

Despite the outcomes of conservative management, many surgeons—especially vascular or microsurgical surgeons—tend to recommend surgical exploration and vascular reconstruction after CRPP for a PPH, with good results reported [10, 14, 15]. Immediate surgical exploration before fracture reduction has even been recommended [15], although disadvantages of the procedure have been reported to include a long scar and the occurrence of re-occlusion and stenosis of the brachial artery [29, 30].

Surgical exploration is recommended for three main reasons: the first is that ligation of an injured brachial artery is considered to be associated with a high amputation rate based on war-time reports [11, 33]. However, these cases are very different to SHFs, in which the arm can be kept alive even with ligation of the brachial artery due to the collateral circulation [20, 27–29]. Two of our cases underwent CRPP nearly 2 days after injury because of neglect of artery compromise and late referral; however, the pulse recovered in 6 hours in both. This confirms the suggestion from previous studies that collateral circulation is sufficient to support the vitality of a PPH following SHFs, even without fracture reduction.

The second reason is the concern that delayed vascular exploration and reconstruction for a PPH following SHFs will lead to compartment syndrome [16], Volkmann ischemic contractures [34], vasomotor instability [35], forearm claudicating [28, 36], cold intolerance [5, 37], thrombus embolization [38], or retarded development of the limb [39]. Blakey’s study showed high incidence of compartment syndrome. However, the cases in Blakey’s study were referred in a mean time of 3 months (4 days to 3 years) after injury, with late diagnosis [16]. Other studies have shown compartment syndrome following SHFs to be associated with over-swelling, delayed fracture reduction, and elbow flexion of more than 90°; absence of radial pulse alone is not an indication for exploration if there are no other signs of ischemia [40–42]. All cases of retarded development involve the lower limb, and are therefore not related to SHFs [39]. Furthermore, the main area of potential growth in the upper limb is not the elbow [43]. Other complications that have been reported such as vasomotor instability, forearm claudicating, and cold intolerance were either from case reports or a single case in a series [5, 28, 35–37].

The third reason for recommending surgical exploration is the lack of long-term follow-up data and sufficient cases to support the superiority of conservative management, which results in an arm relying on collateral circulation only [10, 30, 31]. However, collateral circulation around the elbow after SHFs has been shown to be sufficient to maintain the limb, with good long-term results [6, 18].

Making the decision of whether to perform surgical exploration requires accurate evaluation of the vascular compromise. There are several ways to evaluate such compromise, including arteriography, CFDU, computed tomography angiography, and magnetic resonance imaging, with the first two being used most often in clinical work. Arteriography can reveal the type of vascular compromise such as compression, entrapment, obstruction, or disruption; performed intra-operatively this approach can avoid unnecessary exploration [10]. Pre-operative arteriography has no benefit because it only confirms a known diagnosis and will delay exploration without usually contributing to decisions regarding clinical management [11, 30]. The disadvantages of arteriography include its invasive nature and the risk of hemorrhage at the puncture site, allergic reaction to the contrast agent, and temporary loss of distal pulse [10]. In contrast, CFDU is noninvasive and easily attainable. A comprehensive pre-operative physical examination is sufficient for assessing compromise of the brachial artery [30], but post-operative CFDU is useful for determining the severity of arterial compromise and informing treatment [11]. In our center, we do not perform vascular exploration where CFDU does not show disruption of the brachial artery and perform post-operative CFDU to reassess the vascular status where aggravation of artery compromise occurred during CRPP and to confirm the continuity of the artery. We believe that intra-operative CFDU immediately after CRPP is beneficial for deciding whether to perform surgical exploration. In our practice, vascular exploration is performed if disruption of the artery is revealed by CFDU; otherwise, close observation will be carried out.

A survey by the British Society for Children’s Orthopedic Surgery showed that only 16% of members would perform vascular exploration immediately after CRPP in the case of a PPH [44]. A recent systematic review comparing two different strategies suggested that CRPP should be the first-line approach for SHFs with either a pale or pink pulseless hand. In the case of pale pulseless hands, there is a chance that radial pulse may return after CRPP; otherwise, immediate vascular exploration is strongly indicated. In a PPH, the traditional strategy of close observation should not be revisited as long as there are no signs of deterioration of the vascular status [12].

The present study has some limitations which should be acknowledged, including the retrospective analysis of data, lack of sufficient cases, and lack of results of final and intra-operative CFDU. Future studies are warranted involving larger cohorts recruited from multiple centers, and the development of an animal model would be beneficial to study the collateral circulation of the elbow and long-term effects of ligation of the brachial artery on limb function and development.

## Conclusions

This study demonstrates that the traditional strategy of close observation should be the first choice for treatment of a PPH after CRPP following SHFs unless there are signs of deterioration of vascular status. CFDU is beneficial for assessing vascular compromise and determining treatment strategies. A close observation will be enough once the continuity of the brachial artery is confirmed by CFDU because of the existence of rich collateral circulation around the elbow joint.

## Data Availability

The data and materials in the current study are available from the corresponding author on reasonable request.

## Abbreviations

SHFs: Supracondylar humerus fractures;
PPH: Pink pulseless hand;
CRPP: Closed reduction and percutaneous pinning;
CFDU: Color-flow Duplex ultrasound;

## References

[1] Farnsworth CL, Silva PD, Mubarak SJ (1998). Etiology of supracondylar humerus fractures. J Pediatr Orthop.

[2] Hanlon CR, Estes WL (1954). Fractures in childhood, a statistical analysis. Am J Surg.

[3] Gosens T, Bongers KJ (2003). Neurovascular complications and functional outcome in displaced supracondylar fractures of the humerus in children. Injury.

[4] Griffin KJ, Walsh SR, Markar S, Tang TY, Boyle JR, Hayes PD (2008). The pink pulseless hand: a review of the literature regarding management of vascular complications of supracondylar humeral fractures in children. Eur J Vasc Endovasc Surg.

[5] Choi PD, Melikian R, Skaggs DL (2010). Risk factors for vascular repair and compartment syndrome in the pulseless supracondylar humerus fracture in children. J Pediatr Orthop.

[6] Louahem D, Cottalorda J (2016). Acute ischemia and pink pulseless hand in 68 of 404 Gartland type III supracondylar humeral fractures in children: urgent management and therapeutic consensus. Injury.

[7] Badkoobehi H, Choi PD, Bae DS, Skaggs DL (2015). Management of the pulseless pediatric supracondylar humeral fracture. J Bone Joint Surg Am.

[8] Saglam Y, Tunali O, Akgul T, Dikmen G, Aksoy M, Dikici F (2014). Mid-term results of pediatric vascular injured supracondylar humerus fractures and surgical approach. J Pediatr Orthop B.

[9] Biz C, Sperotto SP, Maschio N, Borella M, Iacobellis C, Ruggieri P (2017). The challenging surgical treatment of closed distal humerus fractures in elderly and octogenarian patients: radiographic and functional outcomes with a minimum follow-up of 24 months. Arch Orthop Trauma Surg.

[10] Luria S, Sucar A, Eylon S, Pinchas-Mizrachi R, Berlatzky Y, Anner H, Liebergall M, Porat S (2007). Vascular complications of supracondylar humeral fractures in children. J Pediatr Orthop B.

[11] White L, Mehlman CT, Crawford AH (2010). Perfused, pulseless, and puzzling: a systematic review of vascular injuries in pediatric supracondylar humerus fractures and results of a POSNA questionnaire. J Pediatr Orthop.

[12] Delniotis I, Delniotis A, Saloupis P, Gavriilidou A, Galanis N, Kyriakou A, Potoupnis M, Tsiridis E, Ktenidis K (2019). Management of the Pediatric Pulseless Supracondylar Humeral Fracture: A Systematic Review and Comparison Study of “Watchful Expectancy Strategy” Versus Surgical Exploration of the Brachial Artery. Ann Vasc Surg.

[13] Schoenecker PL, Delgado E, Rotman M, Sicard GA, Capelli AM (1996). Pulseless arm in association with totally displaced supracondylar fracture. J Orthop Trauma.

[14] Noaman HH (2006). Microsurgical reconstruction of brachial artery injuries in displaced supracondylar fracture humerus in children. Microsurgery.

[15] Salvati S, Settembrini AM, Bissacco D, Dallatana R, Mazzaccaro D, Crippa C, Romano P, Settembrini P (2017). Vascular Injury Due to Humerus Fracture in Pediatric Age: When the Treatment Is Mandatory. Ann Vasc Surg.

[16] Blakey CM, Biant LC, Birch R (2009). Ischaemia and the pink, pulseless hand complicating supracondylar fractures of the humerus in childhood: long-term follow-up. J Bone Joint Surg Br.

[17] Robertson AK, Snow E, Browne TS, Brownell S, Inneh I, Hill JF (2018). Who Gets Compartment Syndrome?: A Retrospective Analysis of the National and Local Incidence of Compartment Syndrome in Patients With Supracondylar Humerus Fractures. J Pediatr Orthop.

[18] Scannell BP, Jackson JB, Bray C, Roush TS, Brighton BK, Frick SL (2013). The perfused, pulseless supracondylar humeral fracture: intermediate-term follow-up of vascular status and function. J Bone Joint Surg Am.

[19] Weller A, Garg S, Larson AN, Fletcher ND, Schiller JR, Kwon M, Copley LA, Browne R, Ho CA (2013). Management of the pediatric pulseless supracondylar humeral fracture: is vascular exploration necessary?. J Bone Joint Surg Am.

[20] Wolfswinkel EM, Weathers WM, Siy RW, Horowitz KS, Hollier LH (2014). Less is more in the nonoperative management of complete brachial artery transection after supracondylar humeral fracture. Ann Vasc Surg.

[21] Tomaszewski R, Wozowicz A, Wysocka-Wojakiewicz P (2017). Analysis of Early Neurovascular Complications of Pediatric Supracondylar Humerus Fractures: A Long-Term Observation. Biomed Res Int.

[22] Cambon-Binder A, Jehanno P, Tribout L, Valenti P, Simon AL, Ilharreborde B, Mazda K (2018). Pulseless supracondylar humeral fractures in children: vascular complications in a ten year series. Int Orthop.

[23] Delniotis I, Ktenidis K (2018). The pulseless supracondylar humeral fracture: Our experience and a 1-year follow-up. J Trauma Acute Care Surg.

[24] Lipscomb PR (1955). Vascular and neural complications in supracondylar fractures of the humerus in children. J Bone Joint Surg Am.

[25] Rang M, Pring M, Wenger D (2005). Rang’s Children’s Fractures.

[26] Skaggs D, Flynn J, Stanley EA, Skaggs DL, Flynn JM (2006). Trauma about the elbow: overview, supracondylar, and transphyseal fractures. Chapter 5: Staying out of Trouble in Pediatric Orthopaedics.

[27] Clement DA (1990). Assessment of a treatment plan for managing acute vascular complications associated with supracondylar fractures of the humerus in children. J Pediatr Orthop.

[28] Shaw BA, Kasser JR, Emans JB, Rand FF (1990). Management of vascular injuries in displaced supracondylar humerus fractures without arteriography. J Orthop Trauma.

[29] Sabharwal S, Tredwell SJ, Beauchamp RD, Mackenzie WG, Jakubec DM, Cairns R, LeBlanc JG (1997). Management of pulseless pink hand in pediatric supracondylar fractures of humerus. J Pediatr Orthop.

[30] Korompilias AV, Lykissas MG, Mitsionis GI, Kontogeorgakos VA, Manoudis G, Beris AE (2009). Treatment of pink pulseless hand following supracondylar fractures of the humerus in children. Int Orthop.

[31] Benedetti Valentini M, Farsetti P, Martinelli O, Laurito A, Ippolito E (2013). The value of ultrasonic diagnosis in the management of vascular complications of supracondylar fractures of the humerus in children. Bone Joint J.

[32] Ramesh P, Avadhani A, Shetty AP, Dheenadhayalan J, Rajasekaran S (2011). Management of acute ‘pink pulseless’ hand in pediatric supracondylar fractures of the humerus. J Pediatr Orthop B.

[33] Hobson R, Rich N (2004). Vascular Injuries of the Extremities. Vascular Surgery Principles and Practice.

[34] Copley LA, Dormans JP, Davidson RS (1996). Vascular injuries and their sequelae in pediatric supracondylar humeral fractures: toward a goal of prevention. J Pediatr Orthop.

[35] Ruch DS, Seal CN, Koman LA, Smith BP (2002). The pink pulseless hand. J South Orthop Assoc.

[36] Pirone AM, Graham HK, Krajbich JI (1988). Management of displaced extension-type supracondylar fractures of the humerus in children. J Bone Joint Surg Am.

[37] Marck KW, Kooiman AM, Binnendijk B (1986). Brachial artery rupture following supracondylar fracture of the humerus. Neth J Surg.

[38] Broudy AS, Jupiter J, May JW (1979). Management of supracondylar fracture with brachial artery thrombosis in a child: case report and literature review. J Trauma.

[39] Friedman RJ, Jupiter JB. Vascular injuries and closed extremity fractures in children. Clin Orthop Relat Res. 1984;(188):112-9. 6467707

[40] Flynn JC, Matthews JG, Benoit RL (1974). Blind pinning of displaced supracondylar fractures of the humerus in children. Sixteen years’ experience with long-term follow-up. J Bone Joint Surg Am.

[41] Mubarak SJ, Carroll NC (1979). Volkmann’s contracture in children: aetiology and prevention. J Bone Joint Surg Br.

[42] Ramachandran M, Skaggs DL, Crawford HA, Eastwood DM, Lalonde FD, Vitale MG, Do TT, Kay RM (2008). Delaying treatment of supracondylar fractures in children: has the pendulum swung too far?. J Bone Joint Surg Br.

[43] Herring JA (2014). Tachdjian’s Pediatric Orthopaedics from the Texas Scottish Rite Hospital for Children.

[44] Malviya A, Simmons D, Vallamshetla R, Bache CE (2006). Pink pulseless hand following supra-condylar fractures: an audit of British practice. J Pediatr Orthop B.

